# A qualitative 5-country comparison of the perceived impacts of COVID-19 on people living with dementia and unpaid carers

**DOI:** 10.1186/s12877-022-02821-1

**Published:** 2022-02-11

**Authors:** Clarissa Giebel, Katarzyna Lion, Maria Mackowiak, Rabih Chattat, P. N. Suresh Kumar, Monica Cations, Mark Gabbay, Wendy Moyle, Giovanni Ottoboni, Joanna Rymaszewska, Adrianna Senczyszyn, Dorota Szczesniak, Hilary Tetlow, Elzbieta Trypka, Marco Valente, Ilaria Chirico

**Affiliations:** 1 Department of Primary Care & Mental Health, University of Liverpool, Liverpool, UK; 2 NIHR ARC NWC, Liverpool, UK; 3 Menzies Health Institute Queensland, Griffith University, Brisbane, Australia; 4 Department of Psychiatry, Wroclaw Medical University, Wroclaw, Poland; 5 Department of Psychology, University of Bologna, Bologna, Italy; 6 IQRAA International Hospital and Research Center, Kerala, India; 7 College of Education, Psychology and Social Work, Flinders University, Adelaide, Australia

**Keywords:** Dementia, Care, COVID-19, Global health, LMIC, Social care

## Abstract

**Background:**

Emerging evidence shows an impact of the COVID-19 pandemic on people living with dementia and informal carers, without any evidence-based global comparison to date. The aim of this international study was to explore and compare the perceived impact of COVID-19 and associated public health restrictions on the lives of people living with dementia and informal carers and access to dementia care across five countries.

**Methods:**

Informal carers and people living with dementia who were residing in the community in the UK, Australia, Italy, India, and Poland were interviewed remotely between April and December 2020. Participants were asked about their experiences of the pandemic and how restrictions have impacted on their lives and care. Transcripts were analysed by researchers in each country using inductive thematic analysis.

**Results:**

Fifteen people living with dementia and 111 informal carers participated across the five countries. Four themes emerged: (1) Limited access and support; (2) Technology and issues accessing remote support; (3) Emotional impact; and (4) Decline of cognitive and physical health reported by carers. Whilst variations were noted, the pandemic has indirectly affected people with dementia and carers across all five countries. The pandemic removed access to social support services and thus increased carer burden. Remote services were not always provided and were very limited in benefit and usability for those with dementia. As a result, carers appeared to notice reduced cognitive and physical health in people with dementia. Particular differences were noted between India and Poland vs. the UK, Italy, and Australia, with less impact on care provision in the former due to limited uptake of support services pre-pandemic based on cultural settings.

**Conclusions:**

The pandemic has amplified dementia as a global public health problem, and people affected by the condition need support to better access vital support services to live well.

**Supplementary Information:**

The online version contains supplementary material available at 10.1186/s12877-022-02821-1.

## Strengths and limitations of this study

- This is the first study to compare the perceived impact of the pandemic on people with dementia and unpaid carers in five countries and provides a global perspective
- Data were collected in the first 9 months since major pandemic restrictions were imposed worldwide.
- The interview topic guide was co-produced with a person with dementia and unpaid carers, and was adapted to the different cultural contexts as part of this international study.
- We only collected data from one low- and middle-income country, India, and thus data lack representativeness of the effects of the restrictions and the pandemic in LMICs.

## Introduction

Considered a global public health concern, dementia affects over 50 million people worldwide [1, 2], with this number consistently rising. Accessing adequate care for the millions of people living with dementia and their informal carers (i.e., family members, friends) has been difficult for many prior to the COVID-19 pandemic [3]. However, the pandemic appears to have created further difficulties for those affected by the condition.

The majority of people living with dementia are residing in the community, yet a greater focus from media during the pandemic has been on older adults with and without dementia residing in long-term care homes. Considering the increased susceptibility of older adults to the virus, care homes and their residents have naturally been affected to a great extent [4]. However, most people living with dementia also require some form of care and support when they live in their own home. This can include support from paid home carers to administer medication, help with getting the person dressed, or preparing a meal, befrienders visiting and chatting with the person with dementia, to activities and support services outside the person’s home. These include day care centers, respite care, peer support groups, and social activities such as walking or dancing groups. These social support services are vital in supporting people with dementia to live well and independently in the community for longer, by providing social engagement as well as cognitive stimulation and physical activity [5].

Considering the very social nature of these activities and pandemic-related restrictions including social distancing and various lockdowns, emerging research indicates how people living with dementia and informal carers have been affected by the pandemic and a lack of access to these once enjoyed support services [6–9]. A recent longitudinal online survey in the UK has shown how social support service usage has significantly declined and only minimally recovered in 2020 [7]. Findings from Germany further highlight how the first lockdown and the pandemic’s restrictions more broadly have impacted on social activities as well as health service utilisation in older adults with cognitive impairment [10]. While these findings indicate already some detrimental impacts on the lives of people affected by dementia, to date there appears to be no comparison across countries regarding the impact of the pandemic on the lives of those living with or caring for someone with dementia. The only cross-country report to date has explored safe visiting guidelines for care homes during the pandemic [11], yet people living with dementia in the community have received less attention.

Social support services and post-diagnostic support structures are not available or easily accessible in all countries. Evidence from lower- and middle-income countries (LMICs) highlights how people living with dementia are predominantly, if not solely, cared for by their family or within their community [12, 13], with care pathways differing greatly across countries [14]. This suggests that the pandemic may affect dementia care to a lesser degree than in high-income countries where more services appear to be utilised. However, emerging findings on the impact of COVID-19 on informal carers of people with dementia in India highlights increased needs since the pandemic [15], but evidence is very limited to date, with particularly no comparison between different cultural settings.

The aim of this international qualitative exploratory study was to explore and compare the perceived impact of COVID-19 and associated public health restrictions on the lives of people living with dementia and informal carers and access to dementia care across five different countries. Our research question was “How has the pandemic and associated public health restrictions impacted on the lives of people living with dementia and unpaid carers globally?”. While research into the pandemic’s impacts is still emerging, to date no study has explored the impacts on people with dementia and carers on an international scale, including the varied impacts between those residing in high- and low- and middle-income countries (LMICs). Burns et al. [16] published an overview of the impact in six European countries, yet this overview lacked evidence. Research conducted early in the UK in April 2020 has highlighted the sizeable impact of the pandemic on dementia care in this European country [6, 17], leading to the comparison with four other countries in Europe and with India. Considering that people with dementia are amongst the most vulnerable in our societies, and are thus more susceptible to the virus due to their age in most cases and their impairments, it is important to understand the indirect effects of the virus on their lives. Despite recent international vaccine rollouts, the rise of new virus variants and uncertainty surrounding the length of protection from vaccination, as well as the possibility of future pandemics, makes it important to capture the experiences of people living with dementia and carers to provide learning and recommendations for improved support.

## Methods

### Participants and recruitment

People living with dementia who were residing in the community and informal carers (family members, friends) who were or have been caring for someone living with dementia residing in Australia, India, Italy, Poland, and the UK were eligible to take part. Participants had to be aged 18 years and over.

Informal carers and people living with dementia were recruited via third sector and social support organisations, social media, existing networks such as the Liverpool Dementia & Ageing Research Forum, local daily care centers, and clinical practices, as well as snowball sampling. Information about the study was placed in internal newsletters for interested participants to contact the lead researcher about taking part. Social support organisations also contacted some members directly via email, allowing for a sharing of information about the study and enquiring whether they wanted to participate.

Ethical approval was obtained from the University of Liverpool Ethics Committee (UK) [Ref: 7626], from Wroclaw Medical University Ethics Committee (Poland) [Ref: KB-366/220], from the Ethic Committee of the University of Bologna (Italy) [Ref: 41453], from the Human Research Ethics Committee at the Griffith University (Australia) [GU Ref No: 2020/488], and from the IQRAA International Hospital & Research Centre institutional ethics committee (India).

### Data collection

The interview guide was co-produced with a person living with dementia, unpaid carers, clinicians, and social support service providers in the UK and can be found in Additional file 1. This was subsequently adapted culturally and translated into Polish, Italian, and Malayalam for data collection in other countries.

In the UK, data were collected in April 2020, when a national lockdown was in place and older and vulnerable adults were told to shield. Data were collected between August and October 2020 in Australia, when most states were slowly easing restrictions. The only exception was that Australia’s second-most populous state, Victoria, was in lockdown at that time (2nd wave) and borders of other states were closed to all people from Victoria. In Italy, data collection took place between November and December 2020, when regions and autonomous provinces of Italy were classified into three areas: red, orange, and yellow ones. According to the Italian Ministry of Health, each area corresponded to different epidemiological risk scenarios and levels, for which specific restrictive measures were taken. In India, there was a nation-wide lock down during the period of data collection with complete restriction of movement across the country as there was no public or private transport including bus/car/train and flight services. Only emergency transport was allowed by issuing special passes from the district collector. All the supermarkets, vegetable and meat shops, stationary shops and malls were closed. Only limited shops were available for shopping from 10 am to 7 pm only. Data were specifically collected in August 2020 in Kerala, India. In Poland, data collection took place between June and August 2020, when restrictions after the first COVID-19 wave were eased, involving partial return to operation of day care centers (albeit with limited numbers of attendees, division into shift groups, and temperature checks).

Participants provided verbal informed consent at the beginning of the interview. Due to social distancing restrictions, data were collected by telephone or via zoom and video chat platforms, and recorded on an audio recorder. Interviews were subsequently transcribed verbatim.

### Data analysis

In each country, transcripts were given separate ID codes with the ID coding and anonymising process starting at ID01. Transcripts were coded by two researchers experienced in qualitative data analysis. We employed inductive thematic analysis [18] to generate codes and themes first individually and then within each country group. Highlighted codes were discussed amongst country teams once all transcripts were coded, with repetitive codes being formed into themes. These were then jointly discussed at virtual team meetings across each country. UK, Australian, and Indian transcripts were coded in English, with Indian transcripts translated. Polish and Italian transcripts were coded in the original language, with specific quotes after joint analysis translated into English.

### Patient and public involvement

Three unpaid carers and one person living with dementia, as well as a number of third sector organisations and support providers for dementia, were involved in designing the study, helped developed the topic guide, interpret the findings, and contributed to the dissemination. All public advisers were active and equal team members and involved in all team meetings and in all stages of the study in the UK. One UK informal carer has been involved in interpreting the international comparison findings.

## Results

### Sample description

A total of 126 participants across five countries were interviewed for this study (50 in the UK; 26 in Poland; 22 in Italy; 12 in Australia; 16 in India). Specifically, 111 unpaid carers and 15 people living with dementia took part (8 from the UK, 5 from Poland, 2 from Australia). Carers were mostly female (*n* = 83, 74.8%), adult children (*n* = 73, 67%), lived separately from their relative with dementia (*n* = 64, 58.7%), and on average 61 (+/− 9.6) years old [Range 36-91]. People living with dementia were relatively equally gender represented (men n = 8, 53.3%) and on average 69 (+/− 9) years old [Range 50-87]. Dementia subtypes amongst people living with dementia and the people carers cared for were mostly Alzheimer’s disease dementia (*n* = 61, 53.5%), followed by mixed and vascular dementia (14.9%; 12.3%), as well as other rarer dementia subtypes including fronto-temporal dementias and Lewy Body dementia (19.3%).

### Qualitative findings

Thematic analysis identified four overarching themes across the interviews: (1) Limited access and support (four sub-themes); (2) Technology and issues accessing remote support (three sub-themes); (3) Emotional impact (two sub-themes); and (4) Decline of cognitive and physical health reported by carers (two sub-themes). Table 1 provides an overview of quotes by theme and sub-theme.

**Table 1:** Overview of quotes by theme and sub-theme

**THEME 1: Limited access and support**	
**Sudden lack of care**	“We felt a bit abandoned because we couldn’t do it anymore [going to the Alzheimer café], it took off that part which stimulated my mum, so we all found ourselves a bit unprepared, honestly ...” **Italy ID05, Female Carer, daughter** “Well, maybe it’s cruel, thanks to COVID and the fact that I worked remotely, for example, I had more time to take care of my mother directly. The care of our private carer was more limited.” **Poland ID03, Female carer, daughter** “Except that I don’t get the breaks that I was, I was able to get, you know coz my services closed down, too, which with everything else. So, it’s very hard for me to get respite now. Because respite means trying to distance myself from my mom and it means I have to find somewhere to go. So, for quite some time the only place I could go into table can sit by the river for a couple of hours, but there’s nowhere else I could go. So yeah, it’s had a negative impact on me too.” **Australia, Female carer, daughter**
**Different impacts on paid home care staff**	„they’re booted and suited as they say; they’ve got gowns, masks, aprons and they’re all going through the they can’t do social distancing because it’s impossible if they’re changing my wife or or dressing her social distancing is not possible or practice but they do their best, they do their best.” **UK ID25, Male carer, Spouse** “When the pandemic broke out, the private caregiver simply left, only me remained. I mean, the neighbors helped us and someone else from the family. But at this moment everything is on me.” **Poland ID18, Male carer, Spouse** “I also had to hire a private caregiver, since before the pandemic she was 4/5 days a week out of home, and now she is at home all day long, I had difficulties and I needed an external support…otherwise the burden was too heavy.” **Italy ID17, female carer, 62y, daughter**
**Medical support over social care**	„During the coronavirus pandemic access to doctors’ care is extremely reduces as well as treating patients with anything different from coronavirus” **Poland ID09, Female carer, Spouse** „After the corona outburst we didn’t face so many changes and problems. During lockdown there was difficulty to buy medicines. But we adjusted. Now there are no more difficulties. We spend most of the time in our house and farmland.” **India ID01, Male carer, Spouse** “the pandemic brought many difficulties with it …It was hard to go to the doctor, even to call him, and every scheduled exam, after months of waiting, has been cancelled.” **Italy ID21, Female carer, daughter**
**Cultural adaptations to dementia care**	„We know about different types of services like day care centres, palliative care centres etc. But we have no such services nearby our home. We didn’t experience any major changes as we are taking care of mother. There is no external help used.” **India ID03, Male carer, Son** „After corona outburst, I (son) take care of mother because other two children who are in abroad can’t come and visit mother. It makes mother sad otherwise there is no big deal.” **India ID07, Male carer, Son**
**THEME 2: Technology and issues accessing remote support**	
**Remote digital support a helpline**	„it was disconcerting to start with because [...] everything just collapsed overnight. Literally overnight. And I found that a bit depressing, I though well what am I going to do now. But with with things like Zoom and Skype and of course the good old telephone, especially Skype and Zoom because it’s visual, you can interact with the people you’re seeing them it’s not just audible. I found that extremely helpful.” **UK ID08, Male person living with dementia** ‘Concerning the video-call, it was helpful to break the routine, since a new person entered the house, even with a call. This was a respite.. My mother was apathetic before. 2 h a week were not that much, but at least it was something different. My mother had a different expression, she seemed at peace.’ **Italy ID02, female carer, daughter** “Not too much for him, but for me the Dementia Australia meetings went to go online. But there is a set time, and the bottom line is that - Is something else is happening? You can’t participate in them and secondly, while their online you’ve got to make certain that the person that you’re caring for if they are not actively engaged, what do they actually up too? And if something is said by another person, that could be construed as upsetting, then you got to do with the person becoming agitated. You know you’ve got no right to say that or whatever. So, I just found them not satisfactory at all.” **Australia, Female Carer, Spouse**
**No replacement of face-to-face support**	“Nothing can replace contact with other people” **Poland ID16, Female carer, Daughter** “there isn’t that kind of human involvement that allows to better involve people, instead, seen on the phone ... they’re very good but it’s not that human contact that brings you to the attention ... and after a while she got tired” **Italy ID01, Female Carer, daughter** „I have 3 days a week where I can sleep to be perfectly honest I don’t do an awful lot when he’s out there I rest, I rest and I read a book. I look at the television programmes that I want to look at that I can’t look at because he’s talking all the way through them, then I read. I’m not allowed to read when he’s here because he just he won’t let me he wants my attention. I devote, not that I’m complaining, but I devote all my time to him and now he is incontinent as well so I have that to deal with. I’m not complaining don’t get me wrong. [...] I take him to to this place where they’re I leave him there and I trust these people, you have to have somewhere where you can trust them.” **UK ID34, Female carer, spouse**
**Difficulties accessing technology**	„[Tele-consultations are] quite difficult to put into everyday practice, as, firstly, she is not fluent in it and moreover she does not like remote options” **Poland ID08, Male Carer, son** „We had some issues with technology…about internet connection and getting familiar with new devices. Switching from physical buttons to the touchscreen was a big challenge for my parent. It can be easy for us, but it is quite difficult for her to adapt to it” **Italy ID21, female carer, daughter**
**THEME 3: Emotional impact of the pandemic**	
**Upset about restrictions and concern over virus transmission**	„The father is not the one who sits at home, so he gets irritated when asked to sit at home during the lockdown. We have difficulty to handle him during lockdown.” **India ID14, Male carer, son** “As we were closed and not able to meet with others, it just felt like prison. It was hard to handle.” **Poland ID01, Female person with dementia** “Now due to Covid I’m afraid of being infected, so I withdraw into myself, I isolate because I have to protect my mum as much as possible.” **Italy ID12, Female carer, Daughter**
**Carer burden**	“I’m psychologically overwhelmed and I need to breathe some air, I really feel the need to go out, to walk with my legs at a fast pace, I need to get some air, then there is also the psychological burden of anxiety because of this virus, as you experience fear, distress of getting sick ... “ **Italy ID04, Female carer, daughter** „And I was a little bit frustrated, not with the fact that the services weren’t there, but this trying to get her to understand was a bit frustrating. I think… just trying to get her to understand why the things were cancelled and she was OK, but she didn’t quite get it obviously.” **Australia, Female Carer, Daughter**
**THEME 4: Deterioration of cognitive and physical health**	
**Faster deterioration of dementia symptoms**	„So because he can’t go out and he can’t walk that’s impacting on his arthritis but its also impacting on his breathing because he’s not exercising his lungs and also of course then the impact on his mental health because of his Alzheimer’s he’s not getting the stimulation that he needs and so therefor he’s becoming more confused, he’s becoming less verbal, he’s losing words a lot quicker than he was and I think when lockdown eventually comes to an end and he can actually go and access groups he won’t be the same man that he was when he first finished those groups. He’ll have deteriorated so much.” **UK ID38, Female carer, daughter** „I was quite worried and quite as it turns out, quite rightly so. I could see her retreating mentally and physically.” **Australia, Female carer, daughter** „Since we have not utilised those service even before corona breakdown closing these centres has not affected us in a significant way. There are no major changes in the condition before and after the lockdown.” **India ID13, female carer, daughter-in-law** “In my opinion, yes, it affected her because she’s now slower than before, she has fewer interests and spends a lot of time in front of the television that she used to do without, and now she watches a lot and, in my opinion, you become very apathetic in front of TV” **Italy ID18, Female carer, daughter** “I can appreciate the importance of this facility now that she is not there, we have to take care of Mom interchangeably with our sister, but although we try, there is a difference. Mom forgets, she doesn’t want to go anywhere, she sleeps a lot, as if she was thinking more slowly.” **Poland ID01, Female carer, daughter**
**Impact on physical health**	“Once she stopped going out, I could see her ability to be mobile diminishing and that will have a severe impact on the quality of life. And probably the same thing would have with my father once he stopped going to the gym. The wastage in his legs is a lot shakier and not nearly as mobile as he was. And mentally he’s withdrawn more. I think he’s probably a little depressed. Not that he would admit it, but I’m pretty sure he is.” **Australia, Female carer, daughter** „Her independence is more and more limited. Moreover, such situations of reduced activity, staying home, giving up and sort of communication via watching television, accelerates her anxiety” **Poland ID08, Male carer, Son** ‘The situation has changed a bit for what concerns my mum’s autonomous movements…she was able to walk alone before, now we are using a rollator as she has not the same stability anymore…but I don’t know if it is lockdown related or just the natural course of the disease’ **Italy, ID05, female carer, daughter**

### THEME 1: limited access and support

#### Sudden lack of care

The pandemic resulted in a sudden lack of access to social support services, including peer support groups, day care centres, and social activities in the community. Informal carers and people living with dementia felt stranded without this support, and felt affected by the sudden withdrawal of the services, due to pandemic-related restrictions. This had an additional impact on carers, as services were no longer offering respite from their relatives with dementia whilst in the past they would usually attend a day care centre or support group, for example.

#### Varied impacts on paid home care staff

Paid home care staff appeared to be affected differently in each country. Different to social support services provided outside of the home, paid home care staff enters the home, thus increasing the risk of potential virus transmission. Where carers accessed paid home care, paid home care continued to be delivered, yet informal carers faced difficult decisions of whether to continue home care or not, or instead take on the additional caring duties for fear of increased virus transmission. Specifically, in Poland, Italy, and the UK, both families and paid carers were afraid of transmitting the virus to the older person, particularly with staff working with different service users. In Australia, the majority of services were not cancelled, rather provided in a modified way. Different service providers implemented different rules, i.e. paid carers from one company were not allowed to take someone with dementia for a walk outside the house. With paid home care little utilised in India, there was no impact to this service noted amongst interviewed carers.

#### Medical support over social care

Accessing health care services for reasons other than COVID-19 was found to be an issue for many people living with dementia and informal carers. This included getting medication in India, including antipsychotics, at the beginning of the pandemic. Medication usage in general emerged most strongly in the Indian context as a means of support for the person living with dementia, as opposed to care with daily activities, for example. In Poland in particular, accessing medical support was considered a bigger issue than accessing outside social support services, as care was provided predominately within the family.

#### Cultural adaptations to dementia care

Caring for someone with dementia varied across some countries and cultures. In India specifically, but also to some extent in Poland, family members were caring for their relatives with dementia. In India, in the region of Kerala where participants were recruited from, they were less likely to access outside social support services. Care was considered a family duty and therefore care should not be passed on to services or other people. As a result, the pandemic seemed to have much reduced impact on the care of people living with dementia and on informal carers in India than it did in countries such as Australia, Italy, and the UK.

### THEME 2: technology and issues accessing remote support

#### Remote digital support a helpline

Not all services provided remote digital support, especially not at the beginning of the pandemic as experienced in the UK and Italy for example. Technology in general was considered a helpline for many though where it was accessible. Peer support group meetings for example could be hosted remotely via Zoom or other platforms and enabled people with dementia and carers, although mostly the latter, to stay connected with one another to a certain extent. However, this caused difficulties for some carers, as no one would be engaging with the person living with dementia whilst carers were attending online support services.

#### No replacement of face-to-face support

Whilst technology was useful in some instances to at least provide some level of support during the pandemic, participants highlighted how remote support was no replacement for face-to-face support. This was particularly the case for social support services such as day care centres, which in pre-pandemic times also offered the informal carer temporary respite from caring. Face-to-face services were therefore considered vital and missed by participants.

#### Difficulties accessing technology

Many carers reported that the person living with dementia would struggle with technology if they had to organise this themselves, both due to digital illiteracy and due to physical impairments of for example holding a tablet. Carers were often required to set up remote meetings for the person living with dementia, with many people with the condition also not being favourable of remote technology. Even when remote meetings were set up by carers, these were sometimes not beneficial as the person living with dementia failed to understand how to engage with remote meetings.

### THEME 3: emotional impact of the pandemic

#### Upset about restrictions and concern over virus transmission

The physical restrictions to participants’ lives and the sudden lack of social engagement with peers, friends, and family had a significant emotional impact. Some participants described how they felt imprisoned by having to stay at home during lockdown. Some people with dementia were described as restless by their relatives, as they were not used to staying indoors all the time, and were thus struggling with the imposed lockdown restrictions. Participants were also worried and upset about the pandemic itself, which was heightened by fear of virus transmission and infection.

#### Carer burden

With services closed down suddenly during the pandemic, only very slowly opening up again during periods of lower infection rates and thus continued poor access, many informal carers reported feelings of burden and stress about the additional caring duties. Services were no longer offering respite for carers, and the need to pick up extra caring duties caused increased levels of carer burden.

Many carers were also frustrated and exhausted to some extent by having to explain to their relative with dementia repeatedly why restrictions are in place and why services were closed. People living with dementia mostly lacked an understanding of the pandemic and its implications. Repeated explaining could also contribute to increase levels of carer burden.

### THEME 4: decline of cognitive and physical health reported by carers

#### Faster deterioration of dementia symptoms

Many carers across Australia, Italy, Poland and the UK noticed how people with dementia seemed to show increased cognitive deterioration, which was linked to the pandemic’s restrictions. This was particularly concerning for some as they were worried about whether their relative with dementia would have deteriorated too much to be able to attend face-to-face services again once these would resume. The lack of services available during the pandemic and thus lack of interaction and social stimulation appeared to generate a general lack of motivation in people living with dementia. This can contribute further to faster cognitive deterioration.

In India however, as most if not all of the care was provided by family members or by paid servants, the pandemic appeared to have had no impact on the level of dementia. Carers expressed that not having accessed social care service prior to the pandemic was linked to no changes in care receipt during the pandemic, as family resided with the person with dementia and could continue providing care. Thus, carers in India did not express any concern over their relative’s faster cognitive deterioration, and did not notice any cognitive or behavioural changes in relation to the pandemic’s restrictions. This was because care continued being provided by the family and therefore support services were not implicated.

#### Impact on physical health

Carers across Australia, Italy, Poland, and the UK equally noted an impact of lockdown and different types of restrictions on people living with dementia’s physical health and mobility. The lack of social engagement in utilising services appeared to lead to poorer physical health and people struggling to walk. Carers raised the issue of poorer physical health in line with poorer mental well-being, highlighting the overall impact that the restrictions appeared to have on people with dementia. Again, as people with dementia in India did not utilise services, no changes in physical health were noted.

## Discussion

This is the first study we know of to show the global impact of the COVID-19 pandemic on the lives of people living with dementia and their informal carers. COVID-19 is not only impacting on the lives of older adults directly by increased susceptibility and mortality [19], but represents a global health problem indirectly by restricting social support. This has negative implications on cognitive and physical health, and emotional well-being, as shown in our study, with varied and fewer impacts on people with dementia living in India.

Whilst the pandemic has impacted to a great extent on people with dementia and carers in all countries, cultural, as well as economic differences and organisational differences in care provision meant that some were affected more than others. Those living in the UK, Australia, Italy, and Poland seemed to be affected by the sudden closure of face-to-face support services to the greatest extent. Indian carers were not affected at all by closures. This was because care was considered mostly a family and cultural duty, so that services were rarely utilised. This is supported by previous research into expectations of dementia care [20–22]. As a result, informal carers in India specifically seemed to experience no increase in levels of carer burden, whereas carers in other countries were impacted as they took on additional caring duties. This was despite care services in general, as in any other country, having been affected in india during the pandemic as well (Garg et al., 2020). Carer burden is high in most informal carers across the globe [23, 24], and social support services can offer a temporary relief from the care role. Thus, the sudden restrictions imposed on socialising and face-to-face contact have been influenced by COVID-19 restrictions.

It is interesting to note that the familial caring responsibilities are not an LMIC versus high-income country observation. Instead, it is a cultural observation which included carers in Poland, albeit Polish carers utilised more day care centres than Indian carers. Research into various LMICs suggests that dementia care is mostly, if not solely, provided by the family [12, 25]. There are a number of reasons for this, including lack of available or affordable social support services, lack of diagnoses of the condition, which is a key to accessing care in the first place, as well as religious and cultural duties [26]. In Colombia for example, it is a legal duty to take care of family members, including their older family members, to alleviate financial pressure off the government. The comparisons we can draw from this study between LMICs and high-income countries are however limited by the fact that only one LMIC was involved as a representative, whilst participants were also recruited only from one region within India, which has vast variations within-country itself. Further research needs to explore the impact of the pandemic on those living with dementia in different countries, including more LMICs, with findigns from individual country-analyses supporting our findings [27]. Nonetheless, these first findings indicate towards variations in the impact of the pandemic relating to attitudes towards dementia care.

In light of the breakdown of the majority of face-to-face support services and lockdowns restricting people to stay in their own homes, connecting digitally with others, including friends, family, peers, and care providers, emerged as a means to stay socially connected in all countries except India. Considering the dementia population however, with the majority of people living with dementia aged 65 years or over, using technology is a challenging issue for this age group generally [28]. Findings from this study support this noted problem, which is further exacerbated in people with dementia due to their cognitive deficits, rendering them less able to utilise and benefit from digital meetings. This supports previous published opinion pieces on how the pandemic is exacerbating the digital divide [29, 30], as well as very limited evidence to date [31]. Arighi and colleagues [31] for example found that younger carers of people with dementia appeared to enable greater access to telehealth services provided by a memory clinic, due to their higher technological skillsets. The benefit of having an unpaid carer to enable access to remote services is further supported by our findings, whilst our study additionally looks at dementia care in the widest sense, without solely focusing on care provided by memory clinics. Gosse and colleagues [32] further highlight the need for virtual care in dementia, albeit focusing solely on helth care provision, not taking into consideration the need for virtual social care during and likely beyond the current pandemic. As part of this, as also raised by Gosse et al. [32], positive service user perspectives are vital. Whilst technology and access may work, if people living with dementia do not beneft from virtual care, especially socail care, then this suggests a need to either provide hybrid models or face-to-face where possible. Whilst services have likely adapted better a year after the pandemic outbreak, further research needs to explore how people living with dementia, and older adults in general, are adapting to facing a remote social world, and the inequalities this brings with it.

This is particularly important as people with dementia were found to deteriorate faster physically and cognitively, due to the lack of cognitive and physical stimulation and not every person with the condition able to access remote technology, or benefit from it. This supports early emerging reports on faster dementia progression [6, 32] as well as the wider negative impacts of the pandemic on people living with dementia [9]. Whilst evidence is very limited, and more longitudinal studies are required, Canevelli and colleagues [33] reported a worsening of cognitive symptoms and increased dependency in people living with dementia in the community in the first few months of the pandemic in Italy. Again, these detrimental impacts were not noted in India, likely due to the familial care responsibilities. However, other research from India has shown a more severe impact of the pandemic on those living with dementia and informal carers [15], which suggests that there may be regional differences and service infrastructure variations which influence these impacts. Considering that India is one of the most populated countries in the world with 1.366 billion residents, variations are likely between more rural, peri-urban, and urban regions. Thus, it is likely that region-specific data collection as part of our study has not captured faster deterioration in Indian participants, whilst clearly evidencing the global impact of the pandemic on dementia symptomatology and thus increased care needs.

### Limitations

While this study benefited from a broad representation of participants across five countries, and the advantage of including data from different time points of the pandemic in five different countries, there were some limitations. As discussed, India is an example of a LMIC, which means that findings are not generalisable for developing countries. India was chosen as the LMIC representative due to known variations in dementia care delivery prior to the pandemic. However, this study does provide the first comparison of the impact of the pandemic in various countries, which needs to be expanded on in future research. As each country had different forms and types of restrictions, people living with dementia and informal carers faced different circumstances. Pandemic-related restrictions were in place to different degrees in all five countries, and data clearly highlights the global impact that these restrictions have had and are having on those affected by dementia. Lastly, whilst the study benefits from a large number of participants, fewer people with dementia took part than unpaid carers. In addition, people with dementia came from three of the five countries – the UK, Poland, and Australia, so that the experiences of people with dementia are restricted to those countries, as opposed to the experiences of unpaid carers about themselves and about their relatives with dementia. Future research needs to provide more in-depth understanding of the impacts of people with dementia from people living with the condition across different countries.

## Conclusions

This exploratory study highlights the global burden of the pandemic on people living with dementia and their unpaid carers, albeit some differences have been noted in the extent of these impacts between cultures and countries. People with dementia and their carers require more support and social care services need to be enabled better to care for those affected by dementia during the ongoing pandemic. The virus continues to affect people due to a lack of equally distributed vaccinations across the globe as well as emerging new variants reducing the efficacy of earlier vaccinations. Thus, whilst further research needs to explore the impact of the pandemic in other countries, including more LMICs, this study clearly highlights how the pandemic has further exacerbated dementia as a global public health issue.

## Supplementary Information

**Additional file 1.** Interview topic guide.

## Data Availability

Data may be obtained from a third party upon reasonable request submitted to the lead author (CG) and if approved by the entire study team.

## Abbreviation

LMICs: Low- and middle income countries

## References

[1] World Health Organisation (WHO) (2017). Global action plan on the public health response to dementia 2017-2025.

[2] Alzheimer’s disease international (ADI) (2020). From plan to impact III: maintaining dementia as a priority in unprecedented times.

[3] Kerpershoek L, de Vugt M, Wolfs C (2020). Is there equity in initial access to formal dementia care in Europe? The Andersen model applied to the Actifcare cohort. Int J eriatric Psychiatry.

[4] Burton JK, Bayne G, Evans C (2020). Evolution and effects of COVID-19 outbreaks in care homes: a population analysis in 189 care homes in one geographical region of the UK. Lancet Healthy Longevity.

[5] Orellana K, Manthorpe J, Tinker A (2020). Day centres for older people: a systematically conducted scoping review of literature about their benefits, purposes and how they are perceived. Ageing Soc.

[6] Giebel C, Cannon J, Hanna K (2021). Impact of COVID-19 related social support service closures on people with dementaia and unpaid carers: a qualitative study. Aging Ment Health.

[7] Giebel C, Pulford D, Cooper C, et al. COVID-19 related social support service closures and mental well-being in older adults and those affected by dementia: a UK longitudinal survey. BMJ Open. 2021. 10.1136/bmjopen-2020-045889. 10.1136/bmjopen-2020-045889 PMC7813330 33455941

[8] Talbot CV, Briggs P. ‘Getting back to normality seems as big of a step as going into lockdown’: the impact of the COVID-19 pandemic on people with early-stage dementia. Age Ageing. 2021. 10.1093/ageing/afab012. 10.1093/ageing/afab012 PMC7929391 33481988

[9] Bascu JDR, O’Connell ME, Webster C, Poole L, Wighton MB, Sivananthan S (2021). A scoping review of COVID-19 experiences of people living with dementia. Can J Public Health.

[10] Thyrian JR, Kracht F, Nikelski A (2020). The situation of elderly with cognitive impirment living at home during lockdown in the Corona-pandemic in Germany. BMC Geriatr.

[11] Low F-F, Hinsliff-Smith K, Sinha S, et al. Safe visiting at care homes during COVID-19: a review of international guidelines and emerging practices during the COVID-19 pandemic. LTCCovid 2021; Last accessed 09/02/2021 https://ltccovid.org/2021/01/19/safe-visiting-at-care-homes-during-covid-19-a-review-of-international-guidelines-and-emerging-practices-during-the-covid-19-pandemic/

[12] Balouch S, Zaidi A, Farina N, et al. Dementia awareness, beliefs and barriers among family caregivers in Pakistan. Dementia. 2020. 10.1177/2F1471301220915066. 10.1177/1471301220915066 32223333

[13] Nankinga PN, Maling S, Chemali Z (2020). Informal support for people with Alzheimer’s disease and related dementias in rural Uganda: a qualitative study. Int J Ment Heal Syst.

[14] Volpe U, Amin H, Ayinde OO (2019). Pathways to care for people with dementia: an international multicentre study. Int J eriatric Psychiatry.

[15] Vaitheswaran S, Lakshminarayanan M, Ramanujam V, Sargunan S, Venkatesan S (2020). Experiences and needs of caregivers of persons with dementia in India during the COVID-19 pandemic – a qualitative study. Am J Geriatr Psychiatr.

[16] Burns A, Lobo A, Rikkert MO, et al. COVID-19 and dementia: experience from six European countries. Int J eriatric Psychiatry. 2021. 10.1002/gps.5497. 10.1002/gps.5497 PMC8014272 33462849

[17] Giebel C, Hanna K, Cannon J (2020). Decision-making for receiving paid home care for dementia in the time of COVID-19: a qualitative study. BMC Geriatr.

[18] Braun V, Clarke V (2006). Using thematic analysis in psychology. Qual Res Psychol.

[19] Imam Z, Odish F, Gill I (2020). Older age and comorbidity are independent mortality predictors in a large cohort of 1305 COVID-19 patients in Michigan, United States. J Intern Med.

[20] Herat-Gunaratne R, Cooper C, Mukadam N (2020). “In the Bengali vocabulary, there is no such word as care home”: caring experiences of UK Bangladeshi and Indian family carers of people living with dementia at home. The Gerontologist.

[21] Kowalska J, Gorączko A, Jaworska L, Szczepańska-Gieracha J (2017). An assessment of the burden on polish caregivers of patients with dementia: a preliminary study. Am J Alzheimers Dis Other Dementias.

[22] Szczesniak D, Droes R, Meiland F (2018). Does the community-based cobined meeting center support programme (MCSP) make the pathway to day-care activities easier for people living wtih dementia? A comparison before and after implementation of MCSP in three European countries. Int Psychogeriatr.

[23] Prince M, Brodaty H, Uwakwe R (2012). Strain and its correlates among carers of people with dementia in low-income and middle-income countries. A 10/66 dementia research group population-based survey. Int J eriatric Psychiatry.

[24] Sutcliffe C, Giebel C, Bleijlevens M (2017). Caring for a person with dementia on the margins of long-term care: a perspective on burden from 8 European countries. JAMDA.

[25] Brooke J, Omorogieva O. Contemporary views on dementia as witchcraft in sub-saharan Africa: a systematic literature review. J Clin Nurs. 2019. 10.1111/jocn.15066. 10.1111/jocn.15066 31531993

[26] Parker M, Barlow S, Hoe J, Aitken LM. Persistent barriers and facilitators to seeking help for a dementia diagnosis: a systematic review of 30 years of the perspectives of carers and people with dementia. Int Psychogeriatr. 2020. 10.1017/s1041610219002229. 10.1017/S1041610219002229 32024558

[27] Roach P, Zwiers A, Cox E, et al. Understanding the impact of the COVID-19 pandemic on well-being and virtual care for people living with dementia and care partners living in the community. Dementia. 2020. 10.1177/2F1471301220977639. 10.1177/1471301220977639 PMC7952494 33381996

[28] Wu Y-H, Damnee S, Kerherve H (2015). Bridging the digital divide in older adults: a study from an initiative to inform older adults about new technologies. Clin Interv Aging.

[29] Seifert A, Cotten SR, Xie B (2021). A double burden of exclusion? Digital and social exclusion of older adults in times of COVID-19. J Gerontol Series B.

[30] Ramsetty A, Adams C (2020). Impact of the digital divide in the age of COVID-19. J Am Med Inform Assoc.

[31] Arighi A, Fumagalli GG, Carandini T (2021). Facing the digital divide into a dementia clinic during COVID-19 pandemic: caregiver age matters. Neurol Sci.

[32] Gosse PJ, Kassardjian CD, Masellis M, Mitchell SB (2021). Virtual care for patients with Alzheimer disease and related dementias during the COVID-19 era and beyond. Can Med Assoc J.

[33] Canevelli M, Valletta M, Blasi MT, et al. Facing dementia during the COVID-19 outbreak. J Am Geriatr Soc. 2020. 10.1111/jgs.16644. 10.1111/jgs.16644 PMC7300919 32516441

